# The Electric Bath

**Published:** 1877-04

**Authors:** 


*The Electric Bath. Its Medical Uses, Effects and Appliance.* By George M. Schweig, M. D., Member of the New York County Medical Society. New York: G. P. Putnam's Sons, 182 Fifth Avenue; 1877, pp. 131. Price $1.00. Buffalo: Peter Paul & Bro.

This little book is eminently practical. Chapter II. describes the mode of construction of the bath tub, indicates the best materials, and batteries, etc. If approximate prices were given, this part of the work would be more complete.

Mode of administration is next considered, showing that the writer knows of what he speaks. Medication of the water is thought to be necessary in some cases, and useless in others. Tartrate of iron and ammonia is recommended to be used in this manner in chlorosis ; extract of malt, alone or in conjunction with iron, in debility and malnutrition ; iodide of potassium for the elimination of lead ; and iodide in chronic articular affections, accompanied by exudation. On page 22 he says: "In cases where paralyzed muscles have lost their faradic irritability, galvanic interruptions are almost indispensible to successful treatment." Concentration of currents, determination of their direction, and giving a shock, are fully described.

In the chapter on physiological effects, the characteristic differences between the bath and other methods of electrization are discussed. On page 43 he says: "The importance of the electric bath as a physiological stimulent and tonic cannot be overrated. I deem it superior in this respect to any other known agent." Farther on he says: "I have never obtained from galvanization of the nervous centres, which I have practiced in a great number of cases, the striking effects on the alimentary processes, which are so uniform a result of the baths." Freedom from pain is claimed as a characteristic of both galvanic and faradic baths *when properly administered.*

Their therapeutical uses are considered under the following heads: "The electric bath as a diagnostic; as an equalizer of the circulation ; as a general counter-irritant; as a general invigorant and tonic. Its hypnotic and sedative influence. Its improvement of irritation. As a prophylactic."

The closing chapter is devoted to special therapeutics and clinical record. In the clinical record is the test of the whole matter, and the result is strongly in favor of the baths as important accessories to other well-known and approved modes of treatment. What the result might be in other hands than those of Dr. Schweig remains yet to be seen. In paralysis, habitual constipation, locomotor ataxia, mercurial stomatitis, cerebral exhaustion and some neuralgias he obtains unusual and brilliant cures. Cases that have long proved rebellious to other modes of treatment have made a good and sometimes rapid recovery; some more slow, under the use of the baths alone, or of the baths in combination with a little medication. Thirty-four cases are given in full, as illustrations. The author's observation of this agent has, he says, extended over a period of two and a half years. But very few failures are recorded, though baths are not administered to all who apply and can pay for them. They are used like other remedies when, and only when, they are indicated by the conditions of the patient. The advantage of the bath over other modes of electrization is claimed to be in its more general application to the peripheral nerves and consequent greater *power* through reflex action. Galvanism and galvano faradism are both employed, but the former is considered of greatest use and importance. The mode of administration of a well-known remedy had been sufficiently successful to entitle it to a more extended trial; and those who desire to use it will find ample directions in this interesting little work to enable them to do so with success.

